# Six1 homeoprotein drives myofiber type IIA specialization in soleus muscle

**DOI:** 10.1186/s13395-016-0102-x

**Published:** 2016-09-05

**Authors:** Iori Sakakibara, Maud Wurmser, Matthieu Dos Santos, Marc Santolini, Serge Ducommun, Romain Davaze, Anthony Guernec, Kei Sakamoto, Pascal Maire

**Affiliations:** 1 INSERM U1016, Institut Cochin, Paris, 75014 France; 2 CNRS UMR 8104, Paris, 75014 France; 3 Université Paris Descartes, Sorbonne Paris Cité, Paris, 75014 France; 4 Division of Integrative Pathophysiology, Proteo-Science Center, Graduate School of Medicine, Ehime University, Ehime, Japan; 5 Laboratoire de Physique Statistique, CNRS, Université P. et M. Curie, Université D. Diderot, École Normale Supérieure, Paris, 75005 France; 6 Nestlé Institute of Health Sciences SA, EPFL Innovation Park, Lausanne, Switzerland

**Keywords:** Six1, Myosin heavy chain, Skeletal muscle, Slow and fast myofibers, Soleus, Pvalb

## Abstract

**Background:**

Adult skeletal muscles are composed of slow and fast myofiber subtypes which each express selective genes required for their specific contractile and metabolic activity. Six homeoproteins are transcription factors regulating muscle cell fate through activation of myogenic regulatory factors and driving fast-type gene expression during embryogenesis.

**Results:**

We show here that Six1 protein accumulates more robustly in the nuclei of adult fast-type muscles than in adult slow-type muscles, this specific enrichment takes place during perinatal growth. Deletion of Six1 in soleus impaired fast-type myofiber specialization during perinatal development, resulting in a slow phenotype and a complete lack of Myosin heavy chain 2A (MyHCIIA) expression. Global transcriptomic analysis of wild-type and Six1 mutant myofibers identified the gene networks controlled by Six1 in adult soleus muscle. This analysis showed that Six1 is required for the expression of numerous genes encoding fast-type sarcomeric proteins, glycolytic enzymes and controlling intracellular calcium homeostasis. Parvalbumin, a key player of calcium buffering, in particular, is a direct target of Six1 in the adult myofiber.

**Conclusions:**

This analysis revealed that Six1 controls distinct aspects of adult muscle physiology in vivo*,* and acts as a main determinant of fast-fiber type acquisition and maintenance.

**Electronic supplementary material:**

The online version of this article (doi:10.1186/s13395-016-0102-x) contains supplementary material, which is available to authorized users.

## Background

Adult skeletal muscles are composed of slow and fast myofiber types, and each skeletal muscle is composed of a stereotyped percentage of myofibers of different subtypes. Myofiber types are characterized by the expression of slow- or fast-type sarcomeric proteins and specific Ca^2+^-handling proteins that modulate its intracellular concentration during the excitation/contraction/relaxation cycle, by their glycolytic and mitochondrial oxidative metabolic properties as well as by their myoglobin content [1–4]. More specifically, the formation of slow or fast sarcomeric units is achieved by the expression of fiber-type specific isoforms of sarcomeric genes such as myosin heavy chain (*MyHCI*, *MyHCIIA*, *MyHCIIX*, *MyHCIIB*), troponin C (*Tnnc1*, *Tnnc2*), troponin I (*Tnni1*, *Tnni2*), and troponin T (*Tnnt1*, *Tnnt3*). Their metabolic properties are determined by glycolytic enzymes (*Gck*, *Aldoa*, *Pfkm*, *Pfkfb1*, *Eno3*) and by mitochondrial oxidative enzymes [5]. Upon motoneuron firing, stimulation intramyofibrillar Ca^2+^ is released from the sarcoplasmic reticulum to trigger muscle contraction through its binding to troponin C, and further activation of myosin heavy chain ATPase. The cytosolic Ca^2+^ reuptake is carried out by the sarcoplasmic reticulum SERCA proteins encoded by slow-type *Atp2a2* and fast-type *Atp2a1* [1–4]. SERCA activity is regulated by sarcolipin (*Sln*), expressed in slow and fast oxidative fibers, by phospholamban (*Pln*) and by myoregulin (*Mln*) expressed in fast fibers, known repressors of SERCA activity [6, 7] through binding to SERCA proteins [8–10]. In fast-type fibers, parvalbumin (*Pvalb*), a Ca^2+^ buffering protein, removes Ca^2+^ efficiently from the cytosol to promote relaxation of the myofiber [11].

In adult skeletal muscle, the expression of fiber type-specific genes is coordinated by transcription factors whose activity is modulated by cascades of signaling pathways connected with the environment; mainly by Ca^2+^ flux induced by motoneuron stimulation, O_2_, hormones and nutrients availability. Slow motoneuron firing leads to sustained low amplitude elevation in intramyofibrillar calcium concentrations able to activate calcineurin, and CamK while fast motoneuron firing leads to brief intramyofibrillar calcium transients of high amplitude that do not activate calcineurin [12, 13]. Activated calcineurin and CaMK increase the activity of NFAT and MEF2 transcription factors leading to slow sarcomeric gene expression [14, 15]. Muscle mitochondrial oxidation activity which is increased during exercise is under the control of PPARβ/δ and PGC1α and PGC1β, two transcriptional coactivators of PPARβ/δ and activators of oxidative metabolism [16–18]. Whereas muscle specific deletion of both PGC1α and PGC1β does not change muscle fiber type [19], muscle-specific deletion of PPARβ/δ leads to an increased number of fast fibers with reduced oxidative capacity [20], while PPARβ/δ ectopic expression in adult myofibers can change both myosin heavy chain content and oxidative metabolism [21]. Upstream regulators controlling fast myofiber phenotypes are HDACs, Sox6 and *Linc-Myh* known to suppress slow-type gene expression in fast myofibers [15, 22–25], MyoD [26] and Six1 [25, 27]. Hif1α regulates the expression of genes coding for enzymes of the glycolysis pathway, but its deletion does not lead to major modification of the expression of fiber-type specific sarcomeric proteins while it impairs metabolic adaptation upon exercise [28]. Little is known however concerning the importance of these fiber-type regulators in the coordinated expression of slow or fast genes during perinatal development, the period when muscle fiber specialization takes place [1, 2]. In particular, the mechanisms presiding at the expression of a single fast Myh gene in hundreds nuclei of a myofiber have not yet been elucidated [25, 29, 30].

Six homeoproteins are major myogenic transcription factors that directly bind to DNA sequences called MEF3s to control embryonic myogenesis [31–34] and genesis of fast-type myofibers [29, 35]. Forced expression of Six1 and its Eya1 cofactor in adult slow myofibers can reprogram adult slow-twitch oxidative fibers toward a fast-twitch glycolytic phenotype [31]. In adult fast-type skeletal muscles, Six1 directly regulates the expression of numerous fast-type muscle genes [25]. Furthermore, Six1 interacts with the central enhancer of the *Myh* fast genes locus, and controls the expression of the fast-type *Myh* genes (*MyHCIIA*, *MyHCIIX*, *MyHCIIB*) [25].

While *Six1* has been detected in soleus (SOL) muscle at the mRNA and protein levels, its physiological role in slow-type muscles has not been explored [25]. Mouse C57bl6N SOL is classified as a slow-type skeletal muscle, composed of approximately 60 % of slow-type/oxydative myofibers and 40 % of fast-type/oxydative myofibers. In this study, we analyzed the phenotypic consequences of Six1 loss in SOL myofibers during mouse perinatal development and in adult. We show that Six1 governs the specification of fast MyHCIIA myofibers and is required for the maintenance of *MyHCIIA* expression.

## Methods

### Animals and ethics statement

Animal experimentation was carried out in strict accordance with the European convention STE 123 and the *French* national charter on the *Ethics* of *Animal Experimentation*. Protocols were approved by the Ethical Committee of Animal Experiments of the Institut Cochin, CNRS UMR 8104, INSERM U1016. Surgery was performed under ketamine/xylazine anesthesia, and all efforts were made to minimize suffering. *Six1*^*flox/flox*^; *HSA-Cre* conditional knockout mice (*cSix1*KO) were obtained by breeding the *Six1*-*LoxP* mice and transgenic mice expressing a CRE recombinase under the control of the human skeletal actin promoter (HSA) [20]. *Six1*^*flox/flox*^; *HSA-Cre-ER*^*T2*^ conditional inducible knockout mice (*ciSix1* KO) were obtained by breeding the *Six1*-*LoxP* mice and *HSA-CRE-ER*^*T2*^ mice [36]. two-month-old *ciSix1* KO males were given intraperitoneal injection of tamoxifen (1 mg per mouse per day; Sigma) for five consecutive days.

### Immunohistochemistry

For Six1 immunostaining, SOL and gastrocnemius plantaris (GP) muscles were embedded in cryomatrix and quickly frozen in isopentane cooled with liquid nitrogen. Cryostat sections (10 μm) were fixed in 4 % PFA and washed in 1× PBS. The sections were treated with Antigen Unmasking Solution (H-3300, Vector Laboratories) at 95 °C for 10 min and washed in 1× PBS for three times. Sections were treated with 1 % H_2_O_2_ solution for 20 min. After three washes in 1× PBS, they were permeabilized with 0.1 % Triton X-100 for 20 min and left for 1 h in blocking solution (1× PBS, 1.5 % goat serum, 0.1 % Triton X-100). Rabbit polyclonal antibodies directed against Six1 (HPA001893, Sigma) (1/100 dilution), and dystrophin (NCL-DYS2, Leica Biosystems) (1/50 dilution) were applied overnight at 4 °C to the treated sections. The next day, after three washes with 1× PBS containing 0.05 % Tween-20, sections were incubated for 1 h with appropriate fluorescent secondary antibodies (Alexa Fluor 594 goat anti-mouse IgG 1/1000 dilution, Invitrogen) and biotynilated secondary antibodies (anti-rabbit IgG 1/200, Vector Laboratories). After three washes with 1× PBS containing 0.05 % Tween-20, samples were incubated in VECTASTAIN Elite ABC Reagent (Vector Laboratories) for 30 min. After three washes with 1× PBS containing 0.05 % Tween-20, samples were incubated with a tyramide solution labeled by Alexa Fluor 488 (Tyramide Signal Amplification kit, invitrogen). After three washes with 1× PBS containing 0.05 % Tween-20, samples were mounted in Vectashield mounting medium.

For determination of Myh isoform expression, SOL and GP muscles were embedded in cryomatrix and quickly frozen in isopentane cooled with liquid nitrogen. Cryostat sections (10 μm) were washed in PBS, permeabilized with 0.1 % Triton X-100 for 20 min and left for 1 h in blocking solution (1× PBS, 1.5 % goat serum, 0.1 % Triton X-100). Rabbit poly-clonal antibodies directed against Laminin (Z0097, Dako) (1/100 dilution), and mouse monoclonal antibodies against MyHCI (NOQ7.5.4D, Sigma) (1/1000 dilution), MyHCIIA (SC-71, Developmental Studies Hybridoma Bank) (1/20 dilution), fast MyHCs (My-32, Sigma) (1/50 dilution) and MyHCemb (F1.652, sc-53091 Santa Cruz Biotechnology, Inc.) (1/20 dilution) were applied overnight at 4 °C to the treated sections. The next day, after three washes with 1× PBS containing 0.05 % Tween-20, sections were incubated for 1 h with appropriate fluorescent secondary antibodies (Alexa Fluor 488 goat anti-rabbit IgG 1/1000 dilution, Alexa Fluor 594 goat anti-mouse IgG 1/1000 dilution, Invitrogen). After three washes with 1× PBS containing 0.05 % Tween-20, samples were mounted in Vectashield mounting medium. For fiber type counting, each MyHC positive fiber was counted in the entire SOL muscle sections, and the number of positive fibers was divided by the total SOL number of fibers.

### SDH/GPDH staining

Fresh-frozen sections were incubated in 0.2 M phosphate buffer (pH 7.6) containing sodium succinate and nitroblue tetrazolium, NBT (N6876, Sigma Aldrich) for 30 min at 37 °C. Sections were then washed with water and mounted in glycerine gelatin medium. GPDH staining was performed by incubation of unfrozen muscle sections with α-glycerol phosphate as described [37]. For quantification of SDH and GPDH staining, the color images were converted to thresholded images at hue (121-208) and brightness (0-140) by a threshold tool of ImageJ software. The area of thresholded images was measured by ImageJ and normalized by the whole soleus muscle area.

### RNA preparation

Soleus muscles were collected from *cSix1 KO* and control mice. Total RNAs were extracted using Trizol Reagent (Invitrogen) according to manufacturer’s instruction.

### cDNA synthesis and qPCR

RNAs were treated with DNase I (Turbo DNA-free, Invitrogen) and were reverse-transcribed with Superscript III kit (Invitrogen) according to manufacturer’s instruction. Reverse transcription was performed with 1 μg of total RNA. Quantitative real time PCR (Light Cycler 480, Roche) was performed using Light Cycler 480 SYBR Green I Master Kit (Roche) according to the manufacturer’s protocols. PCR was performed for 40 cycles of 95 °C for 15 s, 60 °C for 15 s, and 72 °C for 15 s. Gene expression levels were normalized by the expression level of the housekeeping gene *Actb*. The sequences of the oligonucleotides used in this study are given in Additional file 1: Table S1.

### ChIP experiments

GP and *tibialis anterior* (TA) muscles of 2 months old female mice were minced with scissors immediately after harvesting and fixed in 1 % formaldehyde for 10 min. Formaldehyde was quenched by addition of 0.125 M glycine, and muscles were washed twice in PBS. Muscles were then incubated on ice in lysis buffer (10 mM Tris-HCl pH 7.9, 85 mM KCl, 0.5 % NP40, protease inhibitors (Complete, Roche)) for 10 min and homogenized with a mortar and subsequently with a dounce homogenizer. Nuclei were obtained by centrifugation, incubated in SDS lysis buffer (50 mM Tris-HCl pH 8, 10 mM EDTA, 1 % SDS, protease inhibitors) for 10 min, and sonicated in a bioruptor apparatus (Diagenode). The debris was removed by centrifugation. Sonicated DNA was incubated with 1 μg of *Six1* antibodies (HPA001893, Sigma) under rotation at 4 °C overnight. 20 μl of dynabeads protein G (Invitrogen) were added to the lysates and incubated under rotation at 4 °C for 1 h. The beads were washed with low-salt buffer (2 mM EDTA, 20 mM Tris-HCl pH 8, 150 mM NaCl, 1 % TritonX-100, 0.1 % SDS), high-salt buffer (2 mM EDTA, 20 mM Tris-HCl pH 8, 0.5 M NaCl, 1 % TritonX-100, 0.1 % SDS), LiCl buffer (1 mM EDTA, 10 mM Tris-HCl pH 8, 0.25 M LiCl, 1 % NP40, 1 % deoxycholate), and TE buffer (1 mM EDTA, 10 mM Tris-HCl pH 8). DNA was eluted with elution buffer (1 % SDS, 0.1 M NaHCO_3_) containing 0.1 mg/ml proteinase K (Invitrogen) at 62 °C for 2 h, and proteinase K was inactivated by incubation at 95 °C for 10 min. DNA was finally purified with MinElute PCR purification kit (Qiagen). The amount of specific amplified DNA is normalized by input amplification. The sequences of the oligonucleotides used in this study are as follows. Actb, 5′-TGTTACCAACTGGGACGACA-3′ and 5′-ACCTGGGTCATCTTTTCACG-3′; PvalbMEF3_1, 5′-GGAGCCTTTCATGGTGTGAT-3′ and 5′-GGCGTGTGAATCACTTTCCT-3′; PvalbMEF3_2, 5′- GGATGGGGGTGAATGTGATA-3′ and 5′- CTTCCGGTGTCAGGTACTCC-3′.

### EMSA

EMSA was carried out with *Six1* and *Six4* full-length mouse cDNA cloned into the pCR3 vector (Clontech) as previously described [38]. Recombinant mouse Six1 and Six4 proteins were obtained separately with a T7 transcription/translation kit (Promega) and mixed before contact with the DNA. *Myogenin* MEF3 DNA was incubated with recombinant proteins. Competition experiments were performed in the presence of a tenfold and hundredfold molar excess of unlabeled identified *Pvalb* MEF3_1, *Pvalb* MEF3_2, *Myogenin* MEF3, *Myod1*DRR MEF3, or *Myogenin* NFI sites. The sequences of the oligonucleotides used are as follows, the MEF3 consensus sequence is in italic; MyogF 5′-TGG GGG GGC *TCA GGT TTC* TGT GGC GT-3′. MyogR 5′-ACG CCA CAG AAA CCT GAG CCC CCC CA-3′. NF1F 5′-TAT CTC TGG GTT CAT GCC AGC AGG G-3′. NF1R 5′-CCC TGC TGG CAT GAA CCC AGA GAT A-3′. PvalbMEF3_1F, 5′- TGA GCA TCT *GTA ACC TGA* CCC TTG G -3′. PvalbMEF3_1R, 5′- CCA AGG GTC AGG TTA CAG ATG CT-3′. PvalbMEF3_2F, 5′- TGA GTA CCT *GAC ACC GGA* AGG GGA G-3′. PvalbMEF3_2R, 5′- CTC CCC TTC CGG TGT CAG GTA CT-3′. MyodDRRF, 5′- AGT TGG ATC CGG TTT CCA GAG GC -3′. MyodDRRR, 5′- TGA GAC A*GT AAT TTT A*TC CTG CT -3′.

### Plasmids construction

For the construction of the pGL3-*Pvalb*, C57bl6N mouse DNA was first used as a template to clone the 700 bp promoter of *Pvalb* with forward MluI, 5′- GTAACCTGACCCTTGGAAACCAG -3′ and reverse BglII, 5′- CTTGGATGAGCAGAGGCCGGA-3′ primers. This *Pvalb* promoter fragment was subsequently inserted into an MluI-BglII digested pGL3 basic plasmid. For the construction of the pGL3-*Pvalb*mutMEF3-1, *Pvalb*mutMEF3-2 and *Pvalb*doublemutMEF3, the two MEF3 sites of the promoter were mutated as follows; *Pvalb* MEF3-1: 5′GTAACCTGA to 5′CGCGTCTGA; *Pvalb* MEF3-2: 5′GACACCGGA to 5′CTCGAGGGA. All plasmids sequences were confirmed by sequencing.

### Electroporation

In vivo transfections were also carried out on ten-week old C57Bl6N mice. For each experimental conditions three to five TA muscles belonging to different mice were used. Under isoflurane anesthesia, legs were shaved and muscles were pre-treated by injection of a sterile 0.9 % NaCl solution containing 0.4 U of bovine hyaluronidase/μl 2 h before plasmid injection. Two micrograms of Luciferase-expressing vector and one hundred ng of pRL-TK vector (Promega) were introduced into TA muscles of ten-week-old mice by electroporation as previously described [27]. Two weeks following electroporation, electroporated muscles were frozen in liquid nitrogen before processing for Luciferase assays.

### Luciferase assays

Two weeks after electroporation, TA were dissected and frozen in liquid nitrogen before processing. TA were homogenized in Passive Lysis Buffer (Dual-Luciferase Reporter Assay System, Promega) and rotated for 15 min. The homogenate was centrifuged to remove debris, and the supernatant was used for Luciferase activity measurement according to manufacturer’s instruction (Dual-Luciferase Reporter Assay System, Promega).

### Microarrays

After validation of RNA quality with the Bioanalyzer 2100 (using Agilent RNA6000 nano chip kit), 50 ng of total RNA were reverse-transcribed following the Ovation PicoSL WTA System (Nugen). Briefly, the resulting double-strand cDNA was used for amplification based on SPIA technology. After purification according to Nugen protocol, 5 μg of single strand DNA was used for generation of Sens Target DNA using Ovation Exon Module kit (Nugen). 2.5 μg of Sens Target DNA were fragmented and labelled with biotin using Encore Biotin Module kit (Nugen). After control of fragmentation using Bioanalyzer 2100, the cDNA was then hybridized to GeneChip® Mouse Gene 1.0 ST (Affymetrix) at 45 °C for 17 h. After overnight hybridization, the ChIPs were washed using the fluidic station FS450 following specific protocols (Affymetrix) and scanned using the GCS3000 7G. The scanned images were then analyzed with Expression Console software (Affymetrix) to obtain raw data (cel files) and metrics for quality controls. The analysis of some of these metrics and the study of the distribution of raw data show no outlier experiment. RMA normalization was performed using R and normalized data was subjected to statistical tests.

### Preparation of nuclear and cytosolic proteins

SOL and GP muscles of 2, 3, and 12 weeks old mice were frozen with liquid nitrogen and were homogenized with a mortar. Homogenates were fractionated by NE-PER kit (78833, Thermo Scientific) according to manufacturer’s instruction.

### Western blot

Cell or tissue lysates of GP and SOL from *cSix1*KO and control mice (20–40 μg) were denatured in Laemmli buffer, separated by SDS-polyacrylamide gel electrophoresis and transferred to nitrocellulose membrane. Membranes were blocked in 50 mM Tris-HCl pH 7.6, 137 mM NaCl and 0.1 % (v/v) Tween-20 containing 10 % (w/v) skimmed milk or 5 % (w/v) BSA for 1 h at room temperature and incubated overnight at 4 °C with the indicated primary antibodies (Complex I, NADH dehydrogenase, ab14713, Abcam (Cambridge, UK); Complex II, succinate dehydrogenase, ab109865, Abcam (Cambridge, UK); cytochrome bc1 complex, ab110252, Abcam (Cambridge, UK); Complex IV, cytochrome C oxidase, ab14744, Abcam (Cambridge, UK); Complex V, ATP synthase, ab14748, Abcam (Cambridge, UK); hexokinase II, Sc-6521 (Santa Cruz Biotechnology); glycogen synthase 1, CST #3893, Cell Signaling Technology; AS160, 07-741, Millipore; GLUT4, kind donation from Geoffrey Holman, University of Bath); lamin B, sc-6216 (Santa Cruz Biotechnology); β-tubulin, 05-661 (Millipore); Six1, HPA001893 (Sigma). Detection was performed using horseradish peroxidase conjugated secondary antibodies and enhanced chemiluminescence reagent.

### Statistical analysis

All graphs represent mean values ± SEM. Significant differences between mean values were evaluated using two-tailed, unpaired Student’s *t* test (when two groups were analyzed) or one-way ANOVA followed by Student Newman-Keuls test (for three or more groups).

### Microarray data accession number

Microarray data have been deposited in the Gene Expression Omnibus as accession no. GSE50023.

## Results

### Six1 protein subcellular localization in adult myofibers

**Fig. 1:**
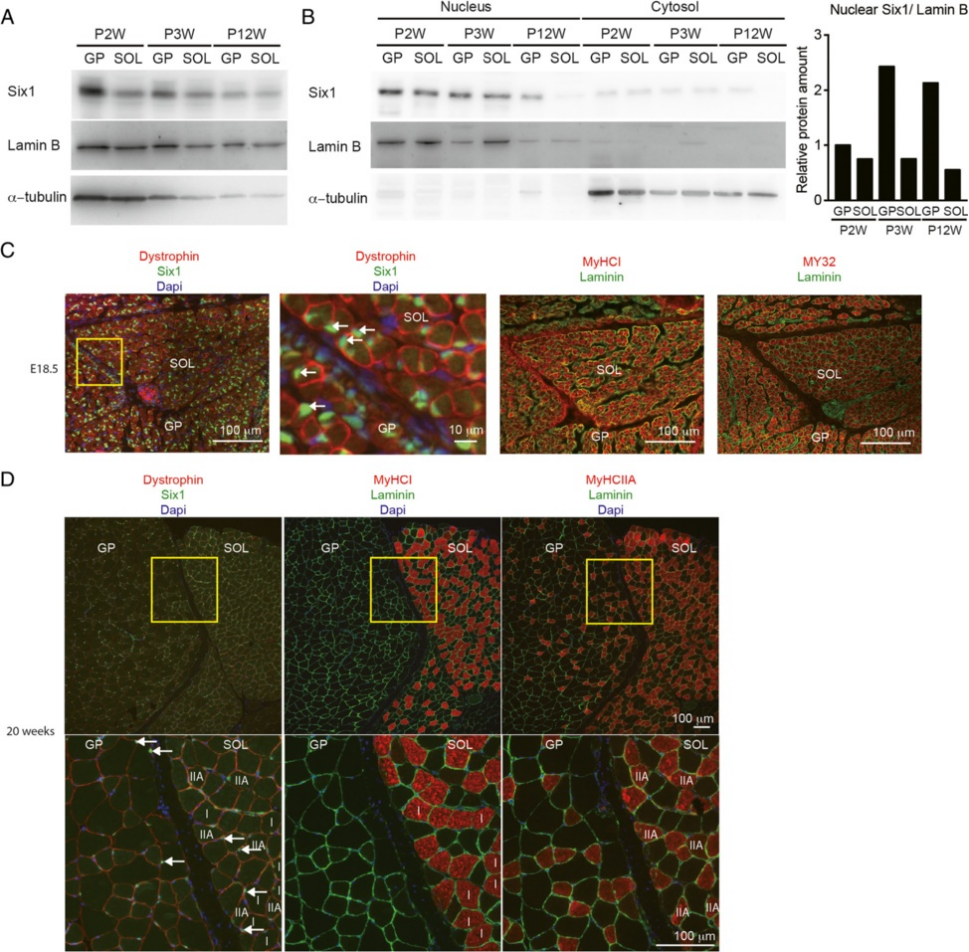
Six1 proteins are predominantly localized in nuclei of fast type skeletal muscles. **a**, **b** Western blot analysis of Six1 proteins in total lysates (**a**) and in nuclear fractions (**b**) of GP and SOL muscles of 2-week, 3-week, and 12-week-old animals. Quantification of nuclear Six1 proteins at these developmental stages is presented. Lamin B is used as a nuclear control protein, and α-tubulin as a cytoplasmic control protein. **c** Immunostaining of Six1 (*green*), Dystrophin (*red*), MyHCI (*red*) Laminin (*green*) and MY32 (*red*) in soleus and gastrocnemius (GP) muscles of embryonic E18.5 mouse fetuses. The second image is an enlargement of the *yellow square* drawn on the first image. *White arrows* indicate Six1 nuclear staining. **d**. Immunostaining of Six1 (*green*), Dystrophin (*red*), MyCHI (*red*), MyHCIIA (*red*) and Laminin (*green*) in SOL and GP of 20-week-old mice. The lower panels are enlargment of the *yellow squares* drawn on the top image. *White arrows* indicate nuclear Six1 detection. Some MyHCI and MyHCIIA positive fibers are indicated as *I* or *IIA*, respectively. *SOL* soleus muscle, *GP* gastrocnemius plantaris muscle

To analyze the properties of Six1 homeoprotein in adult SOL muscles, we first determined its expression pattern. We showed previously that Six1 mRNA accumulates in both fast and slow muscles and that Six1 protein is produced in both type of muscles but accumulates more robustly in the nuclei of fast myofibers as evaluated by immunohistochemistry [25, 27]. We here show that Six1 protein is produced in the GP (fast gastrocnemius and plantaris muscles) and in the SOL of 2 weeks, 3 weeks and adult mice (Fig. 1a), but that the nuclear accumulation of Six1 is drastically reduced between 3 weeks and the adult stage in the SOL (Fig. 1b), although no obvious cytoplasmic Six1 accumulation is observed in the SOL as estimated by Western blot experiments. Six1 protein was further detected in the fast GP and the slow SOL muscles by immunofluorescence using antibodies directed against Six1 at several developmental stages. At embryonic day E18.5, we observed that Six1 is present in the nuclei of myofibers of the GP and SOL expressing at that stage both MyHCI (slow/β) and fast MyHC (detected by My32 antibodies that recognize MyHCemb, MyHCneo and adult fast MyHC) (Fig. 1c). On adult sections, Six1 protein accumulation was detected preferentially in the nuclei of GP as compared with SOL nuclei (Fig. 1d), in agreement with previous results [27]. While GP muscles are mainly composed of myofibers expressing MyHCIIB or MyHCIIX, SOL muscles are composed mainly by myofibers expressing MyHCI (slow/β), MyHCIIA and by few myofibers expressing MyHCIIX. Nuclear Six1 is nevertheless detected in the SOL in the fast MyHCIIA, and in some MyHCI fibers (Fig. 1c). These results showed that in adult hindlimb muscles, Six1 proteins accumulate preferentially in the nuclei of GP fast myofibers than in the nuclei of SOL myofibers (Fig. 1c), in accordance with the low amount of Six1 proteins detected in myonuclei of SOL by Western blot analysis (Fig. 1a), and that this preferential nuclear Six1 accumulation takes place during the perinatal period.

### *Six1* deficiency impairs adult muscle fast type phenotype acquisition in SOL

**Fig. 2:**
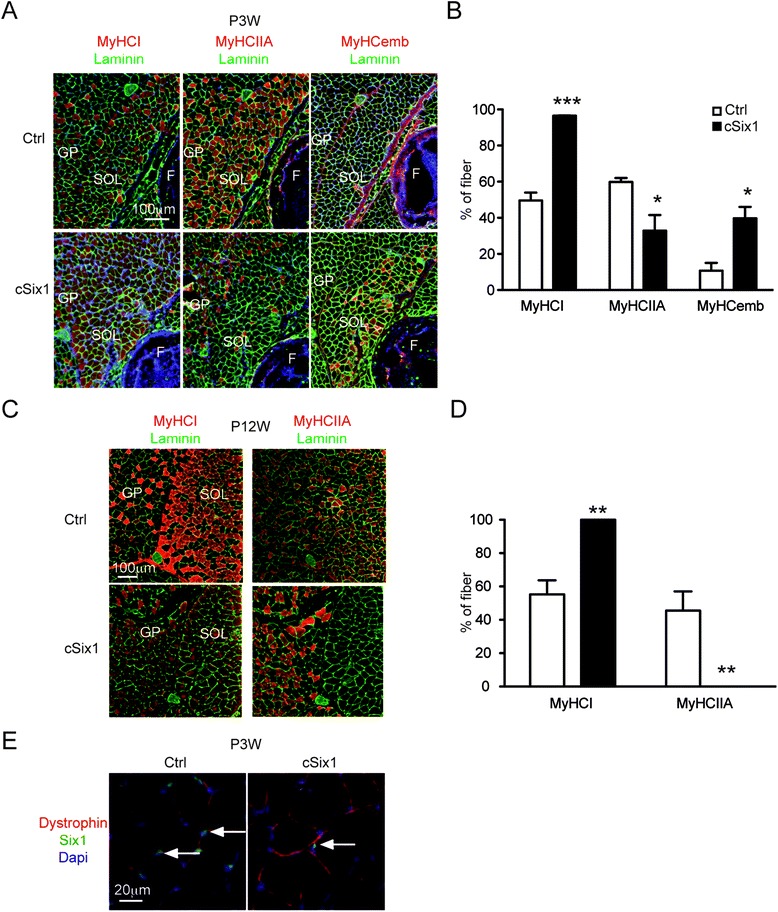
*Six1* deficiency induced a lack of MyHCIIA fibers in soleus. **a** Immunostaining of MyHCI (*red*), MyHCIIA (*red*), MyHCemb (*red*), and laminine (*green*) in soleus muscles of 3-week-old control and *cSix1* KO mice. *SOL* soleus, *GP* gastrocnemius plantaris, *F* fibula. **b** Percentage of myofibers expressing MyHCI, MyHCIIA and MyHCemb in soleus of 3-week-old control and *cSix1* KO mice, Ctrl (*n* = 3), *cSix1* (*n* = 3). **c** Immunostaining of MyHCI (*red*), MyHCIIA (*red*), and laminine (*green*) in soleus of 12-week-old control and *cSix1* KO mice. **d** Percentage of myofibers expressing MyHCI, MyHCIIA in soleus of 12-week-old control and *cSix1* KO mice, Ctrl (*n* = 3), cSix1 (*n* = 3). **e** Immunostaining of Six1 (*green*) and Dystrophin (*red*) in soleus of 3 weeks old *cSix1* KO mice and Ctrl mice. *White arrows* indicate nuclear Six1 staining. Nuclei are stained with Dapi (*blue*). *P < 0.05, **P < 0.01, ***P < 0.001

To characterize the role of *Six1* in adult SOL, we analyzed myofiber-specific Six1 knockout (*cSix1 KO)* mice [25]. *cSix1 KO* mice were viable, and *Six1* mRNA and protein were not detectable in adult GP or SOL muscles [25]. As fiber type specialization that leads to the expression of a single *Myh* gene in all the myonuclei of a given myofiber proceeds during the neonatal stage, we next performed immunohistochemistry to analyze the content of MyHCI, MyHCIIA, and MyHCemb of SOL in 3-week-old *cSix1* mutant myofibers. In control (Ctrl) mice, 10 % of myofibers expressed MyHCemb, 50 % expressed MyHCI, and 60 % expressed MyHCIIA (Fig. 2a, b). In *cSix1 KO* mice, 40 % of myofibers still expressed MyHCemb, 96 % of myofibers expressed MyHCI, and 33 % of myofibers expressed MyHCIIA. This result suggested that sustained expression of Six1 in the perinatal period is important for embryonic to adult fast myofiber transition in SOL and that the decrease of nuclear Six1 accumulation may be important to allow *MyHCI* exclusive expression in slow-type SOL myofibers. It is known that perinatal muscle growth in mice takes place by accretion of new satellite cells (SC) [39]. As Six1 is expressed in SC [40–42], it is possible that Six1 may be transiently expressed by new accreted myonuclei until P21 [39], before its efficient deletion by the HSA-CRE recombinase only active in post mitotic myofibers. To test this hypothesis, we measured Six1 protein accumulation in the nuclei of SOL at 3 weeks of development and detected Six1 positive myonuclei in *cSix1* SOL at this development stage (Fig. 2e). This showed that HSA-CRE recombinase had not yet recombined the *Six1* flox allele in all P21 myonuclei, allowing Six1 to be detected in some nuclei of perinatal myofibers of mutant animals.

In 12-week-old adult animals, at a stage when Six1 is no longer detected in adult *cSix1* myonuclei [25], 100 % of SOL myofibers in mutant mice expressed MyHCI while MyHCIIA was not detectable (Fig. 2c, d), contrary to Ctrl SOL where 45 % of myofibers expressed MyHCIIA and 55 % expressed MyHCI (Fig. 2c, d). In adult *cSix1* mutant SOL, *MyHCIIX* and *MyHCIIA* mRNA became undetectable (Fig. 4b), while the amount of *MyHCI* mRNA increased twice (Fig. 4b).

The number of SOL myofibers present in Ctrl and *cSix1* KO was comparable, excluding that absence of MyHCIIA myofibers in adult mutant animals is the consequence of their death (Additional file 2: Figure S1). We observed no significant modification of the CSA between wt and *cSix1* KO adult myofibers, excluding that Six1 is a main regulator of MyHCIIA myofibers growth in SOL (Additional file 2: Figure S1).

**Fig. 3:**
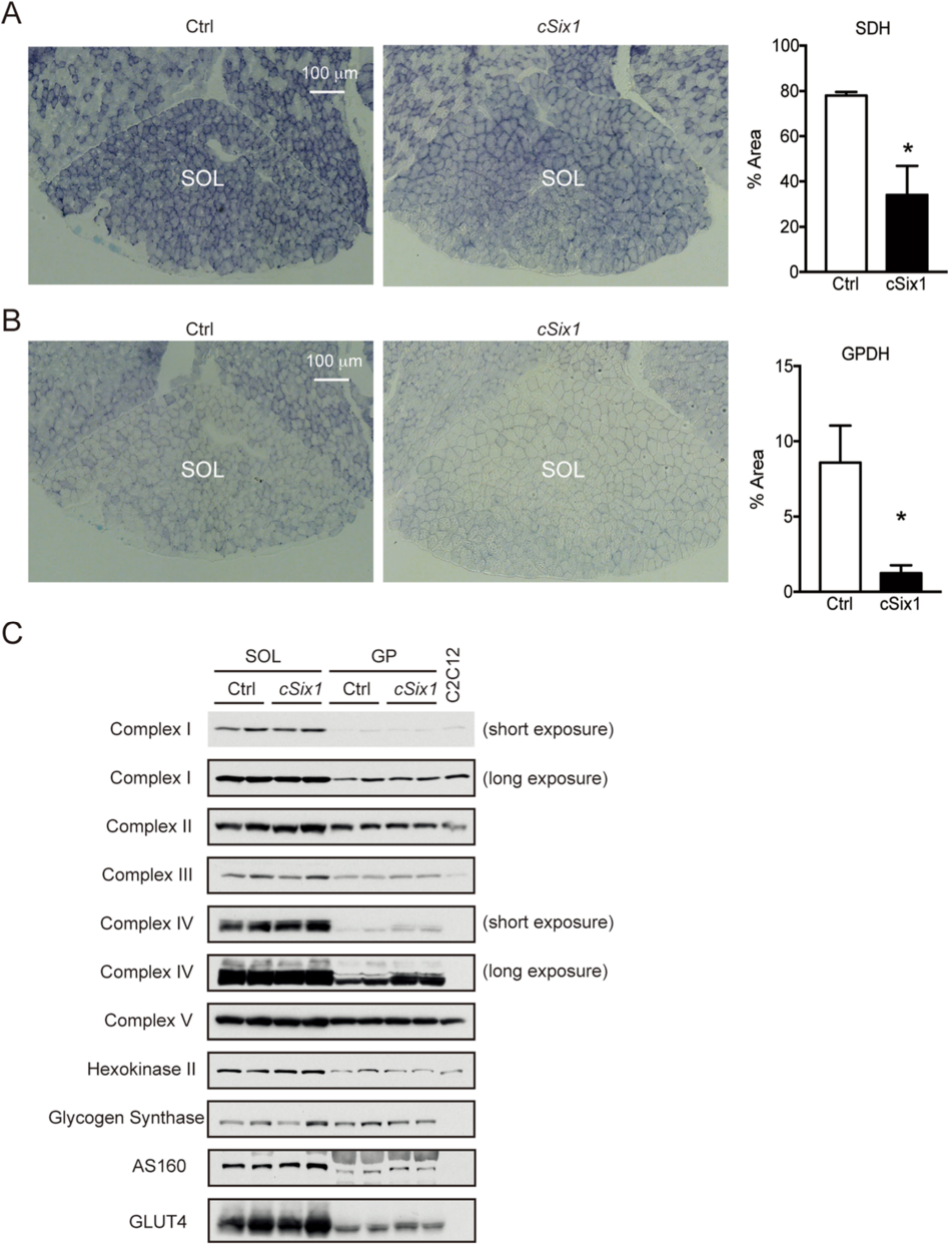
Metabolic properties of *cSix1* KO muscles. **a** SDH staining of soleus of 12-week-old *cSix1* KO and wt mice, and percentage of the area of positive fibers. **b** GPDH staining of soleus of 12-week-old *cSix1* KO and wt mice, and percentage of the area of positive fibers. **c** Western blot analysis of mitochondrial proteins and glucose metabolism proteins in SOL and GP of Ctrl and *cSix1 KO* mice. C2C12, mitochondrial fraction of C2C12

To analyze the oxidative/glycolytic metabolism of mutant SOL muscles, SDH, and GPDH staining were performed on 12-week-old soleus muscle sections (Fig. 3a, b). A two- to threefold decrease of SDH activity was observed in *cSix1* SOL as compared with that of control (Fig. 3a). Nevertheless, no significant difference of mitochondrial protein contents was observed in *cSix1* SOL extracts as determined by Western blots (Fig. 3c). We also observed a robust decrease in GPDH activity in *cSix1* SOL as compared with that of control, suggesting that absence of Six1 decreases glycolytic flux in adult SOL myofibers (Fig. 3b).

### Networks of genes under the control of Six1 in adult SOL

**Fig. 4:**
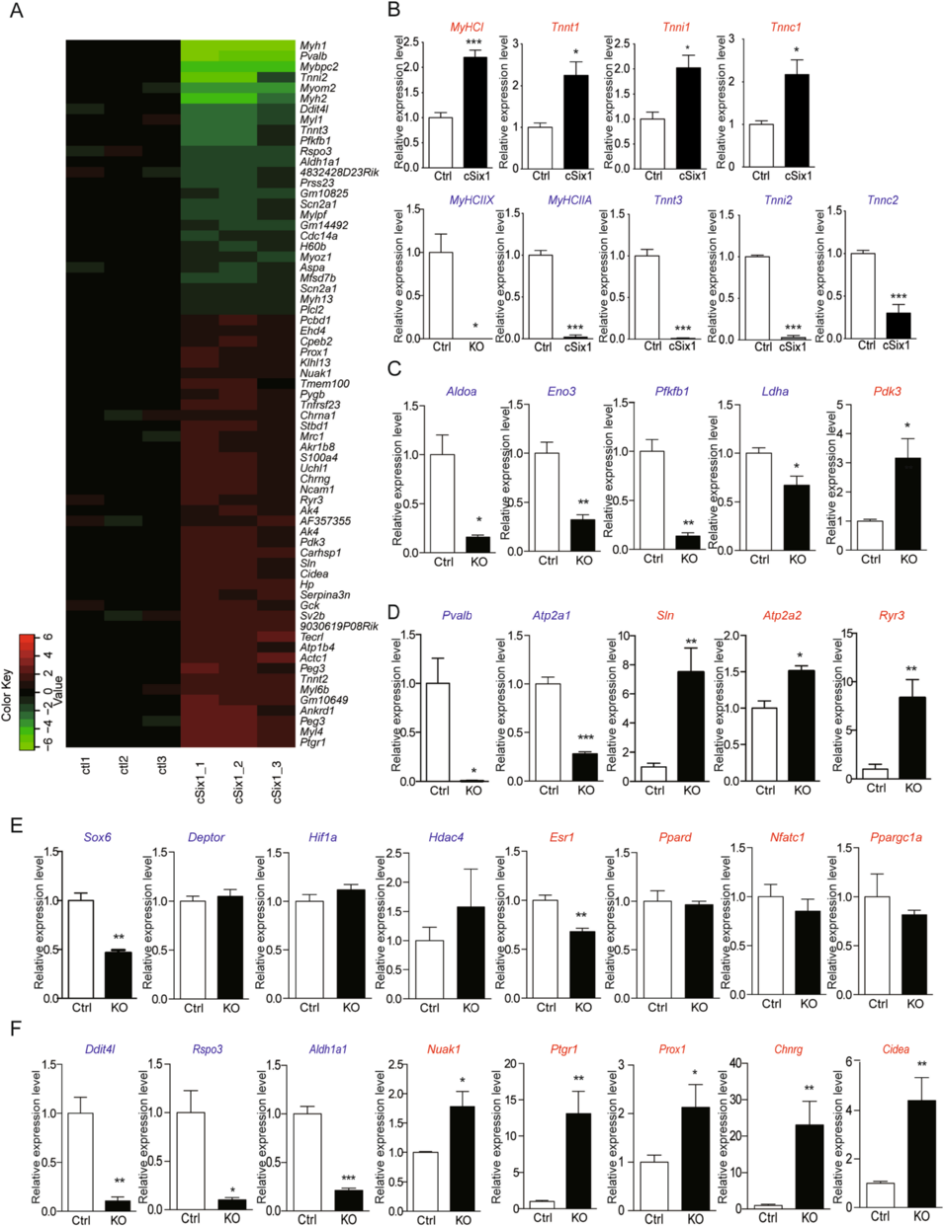
Affymetrix microarray analysis in soleus of *cSix1* mice. **a** Microarray analysis of soleus of 3-month-old *cSix1* KO mice (*n* = 3) and Ctrl mice (*n* = 3): a heat map of genes significantly upregulated (*red*) or downregulated (*green*) by twofold compared with Ctrl mice. **b**–**f** mRNA expression levels of sarcomeric genes (**b**), energy metabolism (**c**), calcium regulation (**d**), transcription factors (**e**), and most down-regulated or up-regulated genes (**f**) in soleus of 3-month-old *cSix1* KO mice, Ctrl (*n* = 4), *cSix1* (*n* = 3). *P < 0.05, **P < 0.01, ***P < 0.001

To explore the networks of genes under the control of Six1 in adult SOL, we performed Affymetrix microarray analyses using RNA from adult Ctrl and *cSix1 KO* mice. Genes whose expression is the most up- or down-regulated (Additional file 3: Table S2) are shown as a heat map and as a bar graph (Fig. 4a and Additional file 4: Figure S2). The expression of several fast-type genes is down-regulated in *cSix1 KO* including *Pvalb*, *Mybpc2*, *MyHCIIA*, *Myl1*, *Myoz1*, and *Mylpf* (Fig. 4a). We validated the expression of those fiber type-specific genes by qPCR. Consistent with immunohistochemistry data, *MyHCIIA* mRNA was no longer detectable in SOL of 12-week-old *cSix1 KO* mice, and *MyHCI* mRNA level was increased by twofold as compared with control (Fig. 4b). The expression levels of slow-type genes (*Tnnt1*, *Tnni1*, *Tnnc1*) were also increased by twofold in *cSix1* KO soleus muscles while the expression of fast-type genes *Tnnt3* and *Tnni2* was not detected and expression of *Tnnc2* was markedly decreased (Fig. 4b). These data show that nuclear accumulation of Six1 observed in SOL MyHCIIA-myofibers is necessary to activate the expression of *MyHCIIA* and of other fast-type sarcomeric genes and to suppress the expression of *MyHCI* and other slow-type muscle genes.

The second most down-regulated gene in *cSix1* SOL is *Pvalb* (Fig. 4a)*,* which is a calcium buffering protein allowing muscle relaxation in fast-type muscles [11, 43–45]. Furthermore, other genes related with calcium regulation were also modified in *cSix1* KO mice (Additional file 4: Figure S2). We validated the down-regulation of *Pvalb* mRNA level in *cSix1* SOL by qPCR experiments (Fig. 4c). The expression level of *Sln*, *Atp2a2*, and *Ryr3* was increased in *cSix1* SOL muscles while the expression of fast-type genes *Myoz1* and *Atp2a1* was strongly decreased (Fig. 4d). These data suggest that Six1 regulates intramyocellular calcium transients through the control of the expression of several fiber type-specific calcium-binding proteins, and suggest a feedback loop between Six1 nuclear accumulation and resting intracellular calcium concentration.

Concerning Affymetrix results, the expression of several genes encoding glycolytic enzymes (*Aldoa, Ldha*, *Pfkfb1*, *PFKm*, and *Eno3*) was also down-regulated (Fig. 4a and Additional file 4: Figure S2 and Additional file 5: Figure S3). *Aldoa* possesses several promoters, and its fast-type one is a known direct target of Six proteins [25, 27]. Muscle-specific *Aldoa* mRNA expression was strongly down-regulated in SOL of *cSix1 KO* mice (Fig. 4c). We also observed the down-regulation of *Slc2a4* (Glut4) and *Tbc1d1*—involved in Glut4 vesicular traffic [46]—of glycolytic genes (*Eno3*, *Pfkfb1*), of muscle *Creatine Kinase*, of *Ldha*, and *Ldhd* of the NAD^+^-dependent isocitrate dehydrogenase *Idh3a*, of *Idh1*, the down-regulation of *Pdp1*, an activator of and the up-regulation of *Pdk3* an inhibitor of pyruvate dehydrogenase activity, and validated by qPCR experiments of their expression (Fig. 4d). No major up- or down-regulation of genes related with oxidative metabolism was detected (Additional file 4: Figure S2), which is consistent with the results of the Western blot analysis showing no major modification of the quantity of the electron transport chain proteins (Fig. 3c).

To investigate potential interaction between Six1 and known fiber-type regulators that may participate downstream of Six1 to the observed phenotype, we analyzed their expression levels by Affymetrix and qPCR (Fig. 4e and Additional file 4: Figure S2). *Sox6* gene, a known repressor of slow sarcomeric genes [22, 24], showed a twofold expression decrease in SOL of *cSix1 KO* mice (Fig. 4e). This down-regulation may be involved in the up-regulation of the expression of its known slow-type gene targets *MyHCI*, *Tnnt1*, *Tnnt2*, *Tnni1*, and *Tnnc1* [22–24, 47] observed in *cSix1* mutant myofibers. Expression of *Esr1* gene (estrogen receptor 1), an activator of the slow/oxidative phenotype in females [48] was decreased in *cSix1 KO* mice. We tested the expression of *Ppargc1a* and *Nfatc1 k*nown transcriptional activators of slow-oxidative genes [1, 2], but found no significant modifications of their expression levels in SOL of *cSix1* mice (Fig. 4e). We were also unable to detect modifications of the expression of mRNA encoding *TFAM*, *PPARβ*, *Rev-erb-α*, or *Mef2C,* known transcription factors controlling the slow/oxidative phenotype of adult myofibers (data not shown). Altogether our results indicate that in SOL of adult mice, Six1 controls the expression of genes coding for fast-type sarcomeric and calcium handling proteins, for glycolytic proteins, and for *Sox6* a known repressor of slow type sarcomeric genes; absence of *Six1* leading to a myofiber switch toward a slower phenotype. Interestingly, in SOL of *cSix1 KO*, we also observed the up-regulation of *Prox1* (Fig. 4f), a known repressor of the fast genes *Tnnt3*, *Tnni2*, *MyHCIIA*, and *Myl1*, and which may also account for their down-regulation [49].

Among the genes showing the most up- or down-regulation in mutant SOL, Affymetrix analysis also revealed a strong reduction of the expression levels of *Ddit4l* (*Redd2*) an inhibitor of the mTOR pathway [50], *Rspo3* (R-Spondin3) and *Aldh1a1* and the increase of the expression level of *Nuak1 (*NUAK family, SNF1-like kinase, 1), *Ptgr1* (Prostaglandin reductase1), *Chnrγ* and *Chnrα1* (Acetylcholin receptor gamma and alpha1), and *Cidea* (cell death-inducing DNA fragmentation factor, alpha subunit-like effector A), an inhibitor of AMPK [51] (Fig. 4a). We also validated the expression of those genes by qPCR (Fig. 4f).

These results indicate that Six1 has a role to enhance the glycolytic pathway in myofibers of adult SOL through the transcription of genes coding for glycolytic proteins, and may modulate more generally glucose utilization in adult myofibers by modulating the expression of key modulators of glucose flux among which Krebs cycle genes (Additional file 5: Figure S3).

### Skeletal muscle-specific Tamoxifen-inducible *Six1* knockout mice also showed fast-to-slow fiber type transition

**Fig. 5:**
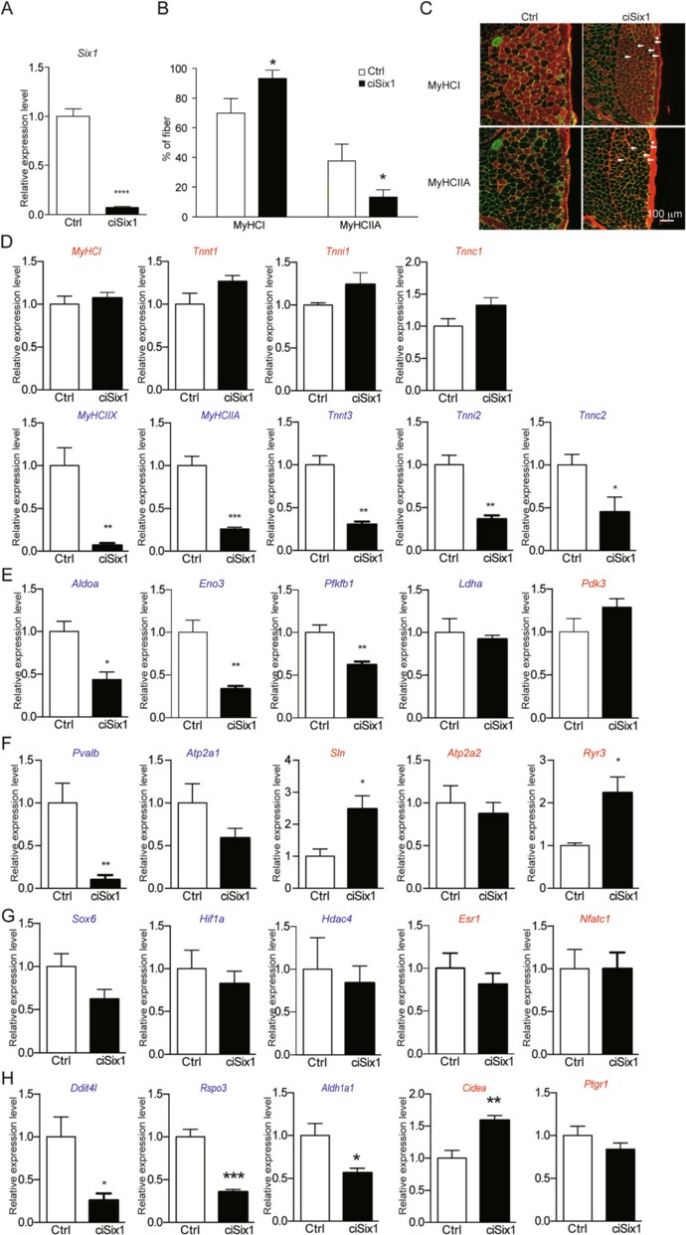
Tamoxifen-induced conditional muscle specific *Six1* knockout reduced fast-type gene expression in soleus. **a** *Six1* mRNA expression levels in SOL of 3 month-old control (Ctrl, *n* = 3) and *ciSix1 KO* (*n* = 4) mice 1 month after tamoxifen injection. **b** Percentage of myofibers expressing MyHCI or MyHCIIA in SOL of 3 month-old control (Ctrl, *n* = 3) and *ciSix1 KO* (*n* = 4) mice 1 month after tamoxifen injection. **c** Immunostaining of MyHCI (*red*) and MyHCIIA (*red*) in SOL of 3 month-old control and *ciSix1 KO* mice 1 month after tamoxifen injection. *Arrows* indicate fibers expressing MyHCIIA, and an *arrow head* indicates a fiber expressing both MyHCI and MyHCIIA in *ciSix1 KO* mice. **d**-**g** mRNA expression levels of sarcomeric genes (**d**), energy metabolism (**e**), calcium regulation (**f**), transcription factors (**g**), and most down-regulated or up-regulated genes (**h**) in SOL of 3 month-old control (Ctrl, *n* = 3) and *ciSix1 KO* (*n* = 4) mice 1 month after tamoxifen injection. *P < 0.05, **P < 0.01, ***P < 0.001

To examine the role of *Six1* in the maintenance of myofibers phenotype in the adult SOL, we bred *Six1*^*flox/flox*^ mice with transgenic mice expressing Cre-ER^T2^ recombinase under the control of the *HSA* promoter [36] to obtain *Six1*^*flox/flox*^; *HSA-Cre-ER*^*T2*^ conditional inducible knockout mice (hereafter named *ciSix1 KO*), to induce *Six1* deletion after Tamoxifen injection. Eight-week-old *ciSix1 KO* mice and their littermate Ctrl were injected with Tamoxifen. One month after Tamoxifen injection, *Six1* mRNA level in *ciSix1 KO* was reduced by ~90 % compared with that in Ctrl mice (Fig. 5a). We further analyzed SOL fiber-type composition by immunohistochemistry (Fig. 5b, c). The percentage of MyHCI positive fiber in *ciSix1 KO* mice increased to 90 %, and the percentage of MyHCIIA positive fiber in *ciSix1* KO mice decreased to 10 %. This result showed that Six1 is required for the maintenance of *MyHCIIA* expression in SOL at the adult stage.

To compare the phenotypic consequences of *Six1* deletion in the adult myofibers and during development, mRNA expression levels of genes identified previously were estimated in SOL of *ciSix1 KO* by qPCR. The expression of fast-type sarcomeric genes (*MyHCIIX*, *MyHCIIA*, *Tnnt3*, *Tnni2*, *Tnnc2*) was also down-regulated (Fig. 5d), consistent with immunohistochemistry results. In contrast, we did not observe a significant up-regulation of slow-type sarcomeric genes (*MyHCI*, *Tnnt1*, *Tnni1*, *Tnnc1*), that may be related to the absence of *Sox6* down regulation (Fig. 5d). For genes controlling glucose metabolism, we observed that the expression level of glycolytic enzymes (*Aldoa*, *Pfkfb1*, and *Eno3*) was *down-regulated*, and that the expression level of *Ldha* and *Pdk3* was not altered (Fig. 5e). Concerning genes controlling calcium handling, mRNA level of *Pvalb* was decreased by 90 % compared with that of Ctrl mice, and an increase of *Sln* and *Ryr3* mRNA levels was observed (Fig. 5f). mRNA level of fiber type regulators such as *Sox6* was not significantly changed between *ciSix1 KO* mice and Ctrl mice (Fig. 5g), contrary to what was observed in *cSix1 KO*. The decreased expression level of *Ddit4l*, *Rspo3*, and *Aldh1a1*, and the increased expression level of *Cidea* were also observed in the SOL of *ciSix1 KO* mice (Fig. 5h). These data show that one main function of Six1 in adult SOL could be to activate the expression of a network of fast-glycolytic specific genes among which are *MyHCIIA*, *Aldoa*, *and Pvalb*. The modulation of slow-type genes expression was not observed in the time window of 4 weeks following Six1 deletion, suggesting that it may take more time to completely transdifferentiate fast-MyHCIIA myofibers into slow myofibers.

### *Pvalb* is a direct target of Six1

**Fig. 6:**
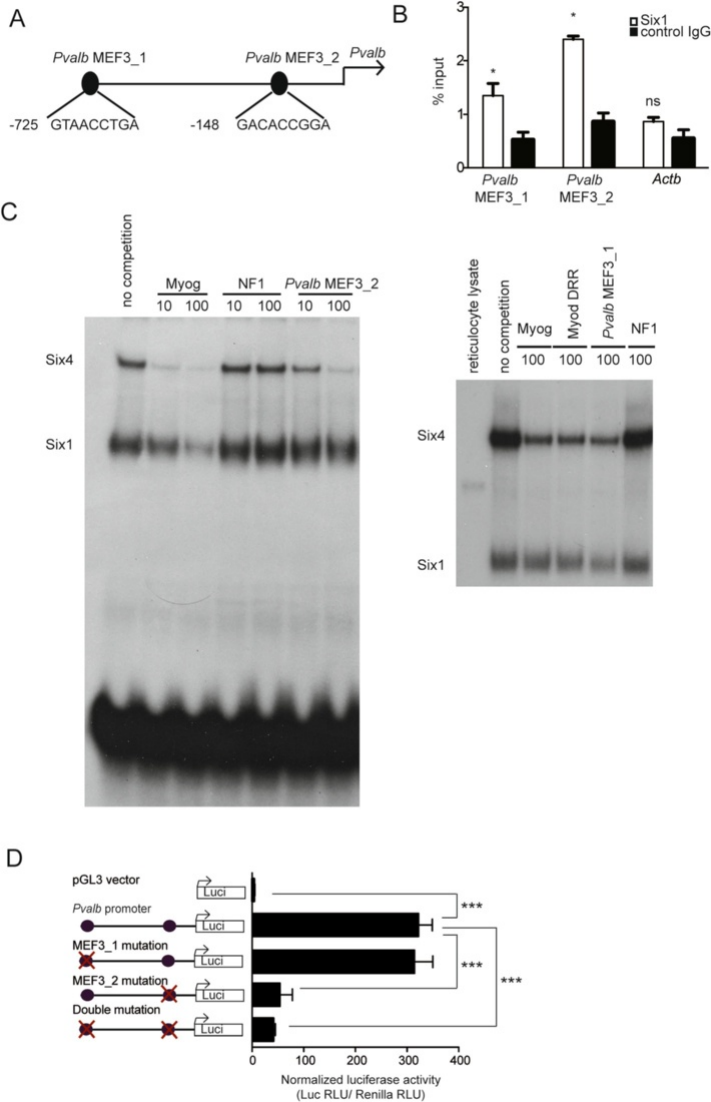
*Pvalb* is a direct target of Six1. **a** Schematic representation of the *Pvalb* promoter. **b** qPCR values of ChIP experiments performed with Six1 antibodies or IgG on GP and TA chromatin, and showing Six1 binding to *Pvalb* MEF3_1 and *Pvalb* MEF3_2. **c** Competitive Electromobility shift assays performed with recombinant Six1 and Six4 proteins and labeled *Myogenin* MEF3 oligonucleotide and 10 or 100 fold molar excess of unlabelled oligonucleotides containing Myogenin MEF3 site, NF1 site, or *Pvalb* MEF3_2 (left panel) and 100-fold molar excess of unlabelled oligonucleotides containing Myogenin MEF3, NF1 site, Myod DRR MEF3 site, or *Pvalb* MEF3_1 sites (*right pannel*) whose sequence is presented on **a. d** Luciferase assays from adult TA muscles electroporated by the indicated luciferase vectors and the TK-renilla luciferase vector allowing normalization. *P < 0.05, ***P < 0.001

A robust reduction of *Pvalb* mRNA observed in *cSix1 KO* mice and in *ciSix1 KO* mice led us to test the hypothesis that *Pvalb* is a direct target of Six1. We analyzed the *Pvalb* promoter sequence and identified MEF3 regulatory elements. Two MEF3 sites located at −725 bp (*Pvalb* MEF3-1) and −148 bp (*Pvalb* MEF3-2) from the transcription start site of *Pvalb* were identified (Fig. 6a). Six1 binding at these MEF3 sites was demonstrated in vivo by ChIP experiments with Six1 antibodies on adult fast GP and TA muscles (Fig. 6b) and confirmed for both of these sites by EMSA assays with recombinant Six1 and Six4 proteins (Fig. 6c). We next tested the transcriptional activating potential of these elements in vivo in transient transfection assays. A 748 bp DNA fragment of the *Pvalb* promoter, including the two identified MEF3 sites, was ligated to pGL3 basic plasmids to generate pGL3-*Pvalb* constructs. Mutations of the two MEF3 sites were introduced in the promoter sequence, giving rise to *Pvalb* mut-MEF3-1, *Pvalb* mut-MEF3-2, and *Pvalb*mut-MEF3-1/2. Luciferase activity was tested after electroporation of these reporter plasmids in adult TA muscles. The activity of a pGL3-*Pvalb* was 100-fold higher than that of the empty pGL3 vector. Luciferase activity was strongly decreased when the MEF3-2 site at -148 was mutated. No further transcriptional decrease of the *Pvalb* promoter was observed when both MEF3 sites were mutated (Fig. 6d). Altogether, these results demonstrate that *Pvalb* is a direct target of Six1, and that Six1 binding to the proximal MEF3 site present in the *Pvalb* promoter is essential for its transcriptional activity in vivo.

## Discussion

In this study, we analyzed the subcellular distribution of Six1 homeoprotein during postnatal development in adult SOL muscle, and the consequences of *Six1* loss during muscle fiber type specialization. We showed that Six1 proteins accumulated differentially in the myonuclei of adult fast and slow fibers. Absence of Six1 delayed the transition from embryonic MyHCemb fiber type to adult fast-type MyHCIIA fiber type, leading to a concerted down-regulation of the fast-type program and up-regulation of the slow-type program. Transcriptomic analysis of Ctrl and mutant SOL identified a network of down-regulated and up-regulated genes upon Six1 deficiency. Particularly, Six1 is required in the SOL to activate the expression of several fast sarcomeric genes, glycolysis genes, fast-type calcium-handling genes as well as *Sox6*, a repressor of slow genes. Conversely, up-regulated expression was observed in *Six1* mutant SOL for numerous slow sarcomeric genes and for *Prox1*, a repressor of fast genes. We further identified *Pvalb*, a key player in intracellular Ca^2+^ buffering, as a direct target of Six1. Altogether our analysis revealed that *Six1* is an essential genetic determinant of fast-type specialization during the post-natal period, and that *Six1* is required in the adult for the maintenance of this phenotype.

### Development of the fast-type IIA phenotype in SOL

Adult mouse SOL is composed of distinct types of myofibers characterized by their contractile and metabolic properties and expressing either the slow *MyHCI*, the fast *MyHCIIA*, or, more rarely, the fast *MyHCIIX* gene [5]. We observed that the Six1 proteins accumulate differentially in adult myonuclei, with a more pronounced enrichment in fast fibers of the GP (Fig. 1) as compared with the fibers of the SOL. However, Six1 mRNA and total protein levels were found similar between SOL and the fast GP [25]. Altogether, these observations suggest that Six1 could be sequestered outside of most MyHCI myonuclei, or actively retained in the nuclei of fast-type fibers, and we show that this control takes place during the perinatal period. Nevertheless, in the SOL Six1 proteins coordinate the expression of genes specific of the adult fast phenotype (*Mybpc2*, *Tnni2*, *Tnnt3*, *Pvalb*, *Sox6*), most probably in MyHCIIA fibers where these genes are coexpressed with *MyHCIIA*. Most of those fast-type genes are also down-regulated in fast GP or TA muscles of *cSix1* mutant, as already reported [25], demonstrating that Six1 acts as a major determinant of fast type gene expression in the different fast fibers subtypes, its absence leading to a “slower phenotype”. In the GP and fast tibialis muscles, absence of Six1 leads to the down regulation of *MyHCIIB* and *MyHCIIX* and the up-regulation of *MyHCI* and *MyHCIIA* [25], while in the SOL it abrogates *MyHCIIX* and *MyHCIIA* expression. To explain this discrepancy one can suggest that the absolute quantity of Six homeoproteins (Six1, Six2, Six4, and Six5) present in the myonucleus tightly controls the expression of the fast *Myh* genes cluster [52]; high levels being required to activate *MyHCIIB*, while lower levels would favor *MyHCIIA* expression. In fast muscles, absence of Six1 would lead to decrease the overall Six level which would nevertheless remain sufficient to activate *MyHCIIA* [25], while in the SOL the threshold of Six proteins reached in absence of Six1 would not allow to maintain *MyHCIIA* expression. Quantification of nuclear Six2, Six4, and Six5 proteins remaining in SOL and GP muscles of Six1 mutant mice may help testing this hypothesis. Alternatively, other yet unidentified specific transcription factors may compensate *Six1* loss in fast TA and GP muscles allowing *MyHCIIA* expression observed in *Six1cKO*, while their absence in SOL would preclude *MyHCIIA* expression. Identification of the transcription factor machinery present specifically in MyHCIIA, MyHCIIX and MyHCIIB myofibers and responsible for the expression of a single fast *Myh* gene at the locus remains to be established. We also observed that in *cSix1* mutant SOL, SDH and GPDH activities are decreased, and that many genes coding for glycolytic proteins are downregulated. More particularly, we identified a decrease of *Phosphofructo-kinase*, of *AldolaseA*, of *Glyceraldehyde-3-phosphate dehydrogenase* and of *Enolase 3* mRNAs, arguing for a decreased glucose use in cSix1 mutant SOL. Furthermore, we observed an increase of *Pdk3* and a decrease of *Pdp1* mRNAs, which should lead to a decrease of Pyruvate Dehydrogenase activity and consequently a decrease of acetyl CoA production. Last, expression of *Isocitrate dehydrogenase 3,* higher in MyHCIIA and MyHCIIX myofibers [5], is also decreased in mutant SOL. Altogether these results suggest a concerted control of sarcomeric genes and of genes controlling glucose metabolism by Six1 homeoproteins.

We observed that 3-week-old animals show already a lower accumulation of Six1 in myonuclei of the SOL as compared to the fast gastrocnemius. Mechanisms underlying Six1 nucleocytoplasmic shuttle and specific accumulation in perinatal and adult fast myofibers remain to be identified. At birth, SOL myofibers express the embryonic *MyHCemb*, neonatal and the slow *MyHCI* genes [53]. Fast *MyHCIIA* is detected at the mRNA level at post-natal stages, and its expression increases during the 3 weeks after birth at the expense of *MyHCemb* and *MyHCneo*, whose expression declines with neuromuscular junction (NMJ) maturation [2]. We observed that in *cSix1* SOL the transition from *MyHCemb* to *MyHCIIA* is impaired, and that three weeks old mutant animals still express *MyHCemb* in the SOL, showing the requirement of Six1 for the transition from embryonic *Myh* to adult *Myh* expression and myofiber specialization.

During post-natal development, three main factors are involved in the emergence of adult myofiber specialization [2] and that may control Six1 accumulation specifically in fast myonuclei.

The first factor involves the influence of slow and fast neuromuscular junctions. Myofibers are still polyinnervated at birth in the SOL, and retraction of polyinnervation only takes place in the first weeks after birth giving rise to slow–twitch fatigue resistant and fast–twitch fatigue resistant alpha motoneurons on slow/MyHCI and fast/MyHCIIA myofibers, respectively [54, 55]. During this period, in the rodents’ SOL, the expression of embryonic and neonatal *Myh* is replaced by the expression of either adult *MyHCI* or *MyHCIIA* [53, 56]. Accordingly, low Six1 nuclear accumulation in MyHCI myofibers may be the consequence of slow motoneurons activity. The main second messenger of slow tonic firing in the myofiber is the Ca^++^ concentration flux that modulates Calcineurin and CaMK activities [57]. Calcineurin phosphatase activity then controls the subcellular localization of NFATc transcription factors [14, 58]. Calcineurin signaling from the perinatal period on appears to be essential for fiber-type specialization, as supported by experiments in transgenic animals showing that its blockade by forced MCIP1 expression impairs slow fibers specialization [59].

In SOL of *Myod1-MCIP1* transgenic mice, *Myoglobin* expression is not reduced, while the expression of *MyHCI* is downregulated from day 7 and is undetected from day 14 [59]. We did not detect in *cSix1KO* or *ciSix1* SOL modification of the expression of *Myoglobin*, suggesting that in both cases mutant myofibers are not completely reprogrammed. Transient Six1 expression in perinatal myofibers of *cSix1* SOL may explain why *MyHCIIA* is transiently expressed in the perinatal period, until the end of SC accretion that takes place in the first 3 weeks post natal [39], and of regular Six1 positive nuclei supply. Thus, the incomplete reprogramming observed in adult *cSix1* mutant SOL may be the consequence of accretion of new satellite cells into the growing post-natal fast MyHCIIA myofibers that provides transiently a genetically or epigenetically programmed fast phenotype that impairs their total reprogramming.

It is known that *MyHCI* expression during the perinatal period as well as in the adult is nerve-dependent [60–62]. Accordingly, all *cSix1* mutant SOL myofibers that express only *MyHCI* should be innervated by slow motoneurons. We observed a strong decrease of the expression of R-Spondin3 (Rspo3) in *cSix1* mutant myofibers. R-spondins are secreted proteins known to enhance Wnt/β-catenin signaling [63] which is a major actor of neuromuscular jonctions (NMJ) [64]. Mice mutant for β-catenin in the myofiber show presynaptic differentiation defects [65]. Whether a Rspo3/β-catenin pathway controlled by Six1 may favor specific stabilization of fast–twitch fatigue resistant alpha motoneuron on future MyHCIIA myofibers is an interesting issue that remains to be tested. Related to this observation, we noted the increase of the expression of *Chnrγ* and *Chnrα1*, suggesting NMJ remodeling in *cSix1* SOL.

A second important mechanism involved in fast myofiber specialization during the perinatal period concerns the influence of thyroid hormone [66, 67]. In agreement, hypothyroidic animals show delayed fast *Myh* transition and prolonged *MyHCemb* expression [68], a phenotype also found in *cSix1* mutant SOL. Affymetrix transcriptomic analysis did not reveal a link between the presence of Six1 in SOL and the level of thyroid receptors (TR) or TR co-factors expression. This suggesting that the absence of fast phenotype acquisition in *cSix1* mutant SOL is not due to a decreased expression of *TR,* although more detailed analysis of the expression of miR-133a1, a direct TR target gene in adult skeletal muscle involved in the control of TEAD1 expression [67], remains to be performed. While Six1 does not control *TR* expression in SOL, the possibility that nuclear Six1 accumulation in fast myofibers could be controlled by the thyroid hormone axis remains an interesting possibility.

A third factor that may participate in myofiber specialization involves intrinsic cell autonomous differences in distinct population of myogenic progenitors leading to myofibers heterogeneity. It is suspected that adult satellite cells associated with slow or fast muscles have intrinsic different genetic properties, although extrinsic factors arising in extracellular matrix or from muscle position and usage in the limb has not been completely excluded [69]. It is therefore tempting to speculate that satellite cells (SC), associated with future MyHCIIA and MyHCI myofibers in the SOL, accreted in the growing myofiber during post-natal development possess specific heritable properties. As myonuclei present in the SOL at 3 weeks show lower Six1 accumulation as compared with myonuclei of the GP, this may suggest that perinatal SC accreted in growing SOL and GP myofibers express different level of Six1, depending of their localization in the niche of MyHCI or subtypes of fast Myh myofibers. It would thus be interesting to test Six1 expression level in GP and SOL associated SC to confirm this hypothesis.

### Fast MyHCIIA expression in adult soleus

In the mammalian genome, a number of genes are organized in clusters such as the *β-globin* [70], the *Hox* [71], and the fast *Myh* cluster [52]. Within these clusters, the precise order of the genes allows their sequential expression through shared enhancers. We showed previously that in adult fast TA and GP muscles Six1 is bound on a central enhancer located at the *Myh* fast locus between *MyHCemb* and *MyHCIIA*, and that it controls the expression of *MyHCIIB* [25]. We show here that in addition Six1 is essential for efficient fiber type shift from *MyHCemb* to *MyHCIIA* in SOL muscles during postnatal development (Fig. 2). The above mentioned fast *Myh* enhancer is able to activate the transcription of the *MyHCIIX*, *MyHCIIA* and *MyHCIIB* genes and may control higher order chromatin conformation at the locus to allow a single fast *Myh* gene to be expressed in all myonuclei of each fiber [25]. *MyHCIIA* activation during the perinatal period in SOL may also depend on this enhancer, and on its own promoter elements known also to interact with Six1 [25, 72].

It will be of major interest to unravel the mechanisms presiding the choice of the expression of a single fast *Myh* gene at the locus in a given myofiber during the perinatal period, its coordinated associated metabolic specialization and the involvement of Six1 in this matter. In the case of the *β*-*globin*, an LCR localized upstream of *Globin* genes controls the spatiotemporal and sequential expression of each gene at the locus. The transcription factor Sox6 binds to each private *Globin* regulatory elements and coordinates the interactions with the LCR through chromatin conformation modification [73, 74]. Interestingly, Sox6 also binds intergenic regions of *Myh* locus in C2 myotubes [22, 24]. An interesting hypothesis would thus be that mechanisms similar to those controlling the *β-globin* locus could act at the *Myh* locus with Six1 and Sox6 cooperating to orchestrate the spatiotemporal expression of the *Myh* fast genes. *Sox6* mRNA is expressed in adult fast type muscles, not in slow type muscles [22], and skeletal muscle specific *Sox6* knockout mice showed fiber type transition from fast to slow. *cSix1* KO SOL mice have an increased expression of slow type genes that may be partly the consequence of decreased expression of *Sox6* mRNA level observed. We have shown previously that Six homeoproteins also control the nuclear accumulation of Sox6 in fetal muscle [29], and we cannot exclude that in *cSix1* KO this is not also the case. It would be interesting to determine whether Six1 is relocalized ouside of the nucleus in adult *Sox6* mutant myofibers since all myofibers in SOL of *Sox6* mutant mice express *MyHCI* [22, 24] and that in transgenic mice overexpressing Sox6 the expression of slow type genes like *MyHCI* and *Tnni1* is down regulated [75]. The consequence of *Sox6* down regulation observed in *cSix1* SOL may also be responsible of *Prox1* up regulation. *Prox1* is a known repressor of fast muscle gene expression in Zebrafish lying downstream of *Sox6* [76] and its deletion in mouse leads to increased expression of fast *MyHCIIA*, *Tnni2*, and *Tnnt3* genes in SOL [49]. Analysis of the consequences of *Prox1* knock down in *cSix1* SOL would allow to test its involvement in the down regulation of *MyHCIIA*, *Tnni2*, and *Tnnt3*.

### *Six* genes redundancy in adult myofibers

In our study, HSA-CREert2 recombinase induced deletion of *Six1* in 2 months adult myofibers led to a switch toward a slow phenotype within 4 weeks following *Six1* deletion. This inducible switch was characterized by the decreased expression of *MyHCIIX*, *MyHCIIA*, *Pvalb*, *AldoA*, and *Eno3* and an increased number of MyHCI positive myofibers. In this model, the up-regulation of slow-type muscle genes 4 weeks after *Six1* deletion was less obvious than observed in HSA-CRE animals, possibly because in this paradigm the expression of *Sox6,* which inhibits a battery of slow muscle genes, is faintly down-regulated. It is possible that during this time period, Six1 direct targets are efficiently down-regulated, while the up-regulation of slow-type genes might require other modifications such as a switch from fast to slow innervation, and/or decrease of *Sox6* expression. Furthermore, the nuclear level of Six4 and Six5 homeoproteins, both expressed in adult myofibers, might be higher in SOL of *ciSix1* than in *cSix1*. In adult muscles, the Six4-Baf60c transcription complex controls *Deptor* expression and transgenic animals with muscle-specific forced expression of Deptor show a switch from oxidative to glycolytic metabolism and are protected from diet-induced insulin resistance [77]. In *cSix1* mutant SOL, the level of *Deptor* mRNA is unchanged, suggesting that the specific Six4/Deptor genetic axis, if maintained in *cSix1* mutant SOL, is sufficient to maintain the level of expression of genes coding for proteins of the glycolytic pathway. *cSix1* SOL presents a severe decrease of GPDH enzymatic activity, which may be correlated to a decreased expression of *Gpd1* in mutant myofibers. Whether further decreased Six activity by combining Six1 and Six4 loss in adult myofibers would lead to increased defects of glycolytic flux remains to be evaluated.

*Pvalb* is a calcium binding protein responsible for fast calcium concentration decrease and relaxation in fast type muscle fiber after excitation. *Pvalb* knockout mice showed prolonged contraction-relaxation cycle [11] and increased fatigue resistance [44] associated with increased mitochondria composition without major modifications of fiber type specific sarcomeric proteins [43]. We demonstrated that *Pvalb* is a direct target of Six1 (Fig. 6), but did not observe up regulation of oxidative metabolism components neither at the mRNA level nor at the protein level (Figs. 3, 4, 5, and 6) in *cSix1* mutant SOL, with on the contrary a decreased SDH activity. Recent study with single fiber proteomics revealed that MyHCI fibers have less mitochondrial proteins and SDH activity compared to MyHCIIA fibers [5]. The loss of MyHCIIA fiber in SOL of *Six1* KO mice might explain the decreased SDH activity observed. In addition, down-regulation of *Esr1* (estrogen receptor 1), which is known to activate mitochondrial oxidation activity in females [48], was also observed in SOL muscles of *cSix1* KO males. These changes may participate in down-regulation of oxidative metabolism in mutant samples (Fig. 3). In that context, down regulation of *Esr1* could cancel the effect of *Pvalb* expression decrease in regard of mitochondrial activity.

## Conclusions

We present the evidence that Six1 homeoproteins are required in mouse SOL muscles for the acquisition of the fast *MyHCIIA* phenotype by controlling *MyHCIIA* expression and the expression of other fast-type muscle genes among which *Sox6*, and by controlling efficient glucose utilization through the control of glycolytic and of Krebs cycle genes.

## Additional files

Additional file 1: Table S1. Sequence of the oligonucleotides used for qPCR experiments. (DOCX 98 kb) Additional file 2: Figure S1. Fiber number and CSA of SOL of 3 months old *cSix1* KO mice. **a** Fiber number in SOL of 3 month-old control (Ctrl, *n* = 4) and *cSix1 KO* (*n* = 3) mice. **b** Average of CSA in SOL of 3 month-old control (Ctrl, *n* = 4) and *cSix1 KO* (*n* = 3) mice. **c** Distribution of CSA in SOL of 3 month-old control (Ctrl, *n* = 3) and *cSix1 KO* (*n* = 3) mice. **P* < 0.05, ****P* < 0.001. (TIFF 816 kb) Additional file 3: Table S2. Affymetrix microarray analysis in SOL of *cSix1* mice. (XLS 7368 kb) Additional file 4: Figure S2. Affymetrix Microarray analysis showing relative gene expression levels of SOL of 3 months old *cSix1* KO mice (*n* = 3) compared with those of Ctrl mice (*n* = 3). A set of genes was selected characteristic of slow/fast sarcomeres, glycolysis, mitochondrial oxidation, transcription factors regulating slow/fast phenotype, Six-homeoproteins-related genes. (TIFF 1290 kb) Additional file 5: Figure S3. Gene coding for the glycolytic pathway and the Krebs cycle are represented. Genes whose expression is modified in *cSix1* KO are indicated as red (up) or green (down). (PDF 281 kb)

## Abbreviations

Aldh1a1: Aldehyde dehydrogenase family 1, subfamily A1
Aldoa: Aldolase A, fructose-bisphosphate
ANOVA: Analysis of variance
Atp2a1: ATPase, Ca^++^ transporting, cardiac muscle, fast twitch 1
Atp2a2: ATPase, Ca^++^ transporting, cardiac muscle, slow twitch 2
Baf60c: Brahma-associated factor 60c
Chnrg: Cholinergic receptor, nicotinic, gamma polypeptide
Cidea: Cell death-inducing DNA fragmentation factor, alpha subunit-like effector A
ciSix1: *Six1*^*flox/flox*^
*HSA-Cre-ER*^*T2*^: Conditional inducible knockout mice
CRE: Cre recombinase
cSix1: *Six1*^*flox/flox*^
*HSA-Cre*: Human skeletal actin–CRE conditional knockout mice
Ctrl: Control mice
Ddit4l: DNA-damage-inducible transcript 4-like
EDL: Extensor digitorum longus muscle
EMSA: Electrophoretic mobility shift assay
Eno3: Enolase 3, beta muscle
Esr1: Estrogen receptor 1 (alpha)
Eya1: EYA transcriptional coactivator and phosphatase 1
Gck: Glucokinase
GP: Gastrocnemius plantaris muscle
GPDH: Glycerophosphate dehydrogenase
Hdac: Histone deacetylase
HSA: Human skeletal actin promoter
Ldha: Lactate dehydrogenase A
MCIP1: Myocyte-enriched calcineurin-interacting protein-1
MEF2: Myocyte enhancer factor 2
MEF3: Myocyte enhancer factor 3
MRFs: Myogenic regulatory factors
Mybpc2: Myosin binding protein C, fast-type
Myh: Myosin heavy chain
MyHC: Myosin heavy chain
Mylpf: Myosin light chain, phosphorylatable, fast skeletal muscle
Myoz1: Myozenin 1
NFAT: Nuclear factor of activated T cells
Nuak1: NUAK family, SNF1-like kinase, 1
PBS: Phosphate buffered saline
Pdk3: Pyruvate dehydrogenase kinase, isoenzyme 3
PFA: Paraformaldehyde
Pfkfb1: 6-phosphofructo-2-kinase/fructose-2,6-biphosphatase 1
Pfkm: phosphofructokinase, muscle
Pgc1α: Peroxisome proliferative activated receptor, gamma, coactivator 1 alpha
PPARβ/δ: Peroxisome proliferator activator receptor delta
Prox1: Prospero homeobox 1
Ptgr1: Prostaglandin reductase 1
Pvalb: Parvalbumin
Rspo3: R-spondin 3
Ryr3: Ryanodine receptor 3
SDH: Succinate dehydrogenase
SEM: Standard error of the mean
SERCA: Sarcoendoplasmic reticulum calcium transport ATPase
Six1: Sine oculis-related homeobox 1
SOL: Soleus muscle
Sox6: SRY (sex determining region Y)-box 6
TA: Tibialis anterior
TFAM: Transcription factor A, mitochondrial
TK: Thymidine kinase
Tnnc: Troponin C
Tnnt: Troponin T
