# Supplementary material for: Six1 homeoprotein drives myofiber type IIA specialization in soleus muscle
Source: Skelet Muscle. 2016 Sep 5;6(1):30. doi: 10.1186/s13395-016-0102-x (PMC5011358; doi:10.1186/s13395-016-0102-x)
Supplement: Additional file 1: Table S1. — Sequence of the oligonucleotides used for qPCR experiments. (DOCX 98 kb) [file 13395_2016_102_MOESM1_ESM.docx]

**Table S1.** Sequences of the oligonucleotides used for qPCR experiments.

| gene name | Forward (5'- 3') | Reverse (5'- 3') |
| --- | --- | --- |
| *Six1* | CTTTAAGGAGAAGTCTCGGG | TTCCAGAGGAGAGAGTTGAT |
| *Myh7* | AGGGCGACCTCAACGAGAT | CAGCAGACTCTGGAGGCTCTT |
| *Myh2* | CCAAGAAAGGTGCCAAGAAG | CGGGAGTCTTGGTTTCATTG |
| *Myh1* | CGGTGGTGGAAAGAAAGG | CAGGAGTCTTGGTTTCATT |
| *Tnnt1* | CCCCCGAAGATTCCAGAAGG | TGCGGTCTTTTAGTGCAATGAG |
| *Tnnt3* | GGAACGCCAGAACAGATTGG | TGGAGGACAGAGCCTTTTTCTT |
| *Tnni1* | ATGCCGGAAGTTGAGAGGAAA | TCCGAGAGGTAACGCACCTT |
| *Tnni2* | AGAGTGTGATGCTCCAGATAGC | AGCAACGTCGATCTTCGCA |
| *Tnnc1* | GCGGTAGAACAGTTGACAGAG | CCAGCTCCTTGGTGCTGAT |
| *Tnnc2* | ATGGCAGCGGTACTATCGACT | CCTTCGCATCCTCTTTCATCTG |
| *Aldoa* | actctctgctgaccgggctct | aatgcttccggtggactcat |
| *Eno3* | CACAGCCAAGGGTCGATTCC | CCCAGGTATCGTGCTTTGTCT |
| *Pfkfb1* | ATGAGCTGCCCTATCTCAAGT | GTCCCGGTGTGTGTTCACAG |
| *Ldha* | TGTCTCCAGCAAAGACTACTGT | GACTGTACTTGACAATGTTGGGA |
| *Pdk3* | TCCTGGACTTCGGAAGGGATA | GAAGGGCGGTTCAACAAGTTA |
| *Pvalb* | ATCAAGAAGGCGATAGGAGCC | GGCCAGAAGCGTCTTTGTT |
| *Atp2a1* | TGTTTGTCCTATTTCGGGGTG | AATCCGCACAAGCAGGTCTTC |
| *Sln* | GGTCCTTGGTAGCCTGAGTG | CGGTGATGAGGACAACTGTG |
| *Atp2a2* | GAGAACGCTCACACAAAGACC | CAATTCGTTGGAGCCCCAT |
| *Ryr3* | CGAGGGACTTGGGAATCGC | CTTGCAGTGCTCTGACAGATAA |
| *Sox6* | cagcgttctgcatctcagc | tctcctccagcttcttctgc |
| *Deptor* | GACGGCGATAAAACTCATGCA | CCTTGTGCTCATCACACACGT |
| *Hif1a* | ACCTTCATCGGAAACTCCAAAG | ACTGTTAGGCTCAGGTGAACT |
| *Hdac4* | CACTGCATTTCCAGCGATCC | AAGACGGGGTGGTTGTAGGA |
| *Esr1* | CCCGCCTTCTACAGGTCTAAT | CTTTCTCGTTACTGCTGGACAG |
| *Ppard* | TCCATCGTCAACAAAGACGGG | ACTTGGGCTCAATGATGTCAC |
| *Nfatc1* | GACCCGGAGTTCGACTTCG | TGACACTAGGGGACACATAACTG |
| *Ppargc1a* | TATGGAGTGACATAGAGTGTGCT | CCACTTCAATCCACCCAGAAAG |
| *Ddit4l* | ATTTGAAGAGACAACATGCCAGA | AGACCTTAGAGCAACCAAGTTTG |
| *Rspo3* | ATGCACTTGCGACTGATTTCT | GCAGCCTTGACTGACATTAGGAT |
| *Aldh1a1* | TGTGGGAATACCGTGGTTGTC | GTGAAGAGCCGTGAGAGGAG |
| *Nuak1* | TCCAACCTGTACCAGAAGGAC | GGGCATCGTTCCATAAATGAGA |
| *Ptgr1* | GAAGGCTTCCCTACGGACG | GGCTGCAACTCTCATGTAAGGA |
| *Prox1* | AGAAGGGTTGACATTGGAGTGA | TGCGTGTTGCACCACAGAATA |
| *Chnrg* | GGCCAGAGACCTCATCTCCT | GGGGTCGTAGTTTCGCATCA |
| *Cidea* | TGACATTCATGGGATTGCAGAC | GGCCAGTTGTGATGACTAAGAC |
| *Actb* | GGCTGTATTCCCCTCCATCG | CCAGTTGGTAACAATGCCATGT |
