# Supplementary figures and images for: Six1 homeoprotein drives myofiber type IIA specialization in soleus muscle
Source: Skelet Muscle. 2016 Sep 5;6(1):30. doi: 10.1186/s13395-016-0102-x (PMC5011358; doi:10.1186/s13395-016-0102-x)

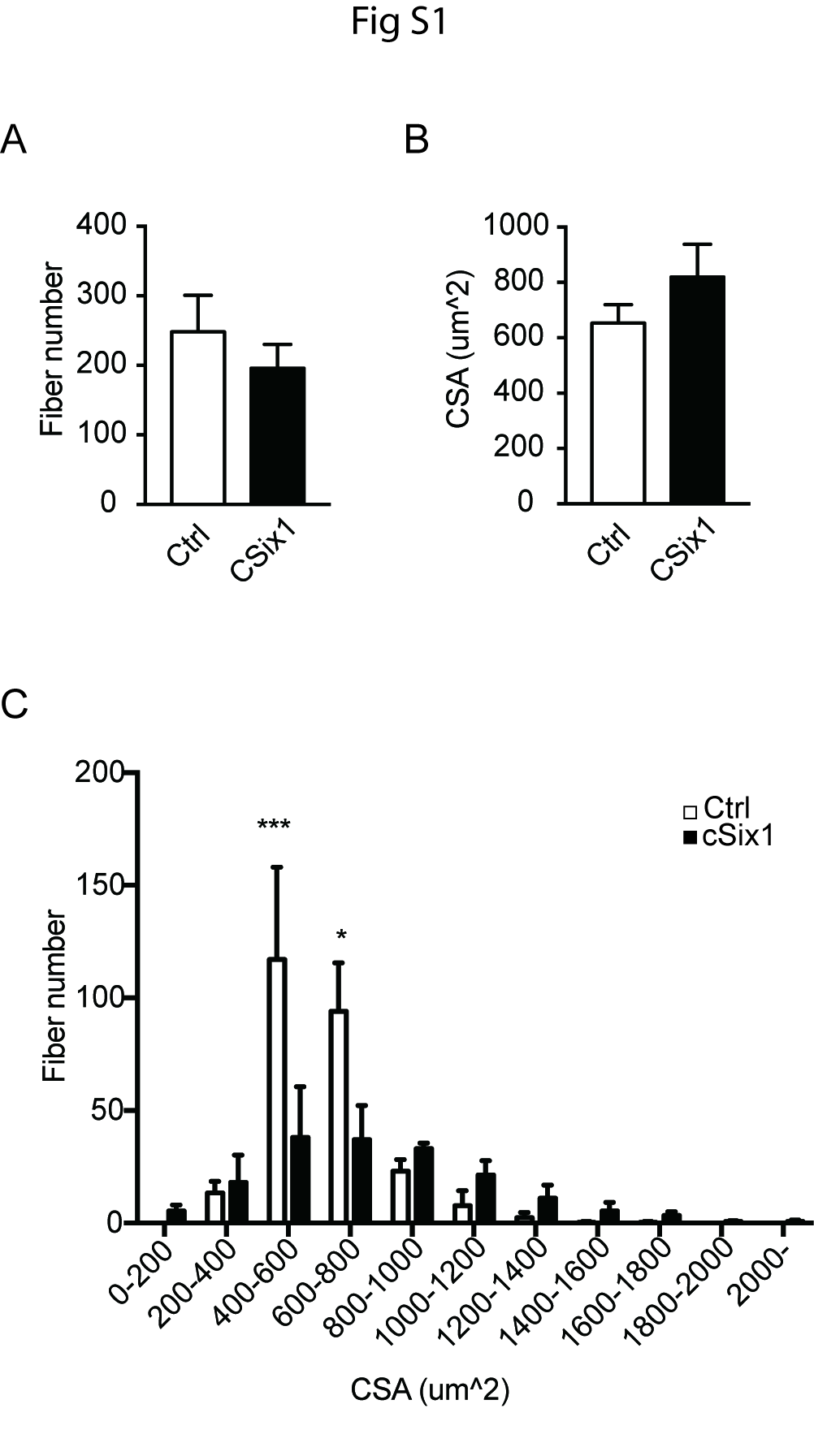

Supplement: Additional file 2: Figure S1. — Fiber number and CSA of SOL of 3 months old cSix1 KO mice. a Fiber number in SOL of 3 month-old control (Ctrl, n = 4) and cSix1 KO (n = 3) mice. b Average of CSA in SOL of 3 month-old control (Ctrl, n = 4) and cSix1 KO (n = 3) mice. c Distribution of CSA in SOL of 3 month-old control (Ctrl, n = 3) and cSix1 KO (n = 3) mice. *P < 0.05, ***P < 0.001. (TIFF 816 kb) [file 13395_2016_102_MOESM2_ESM.tiff]

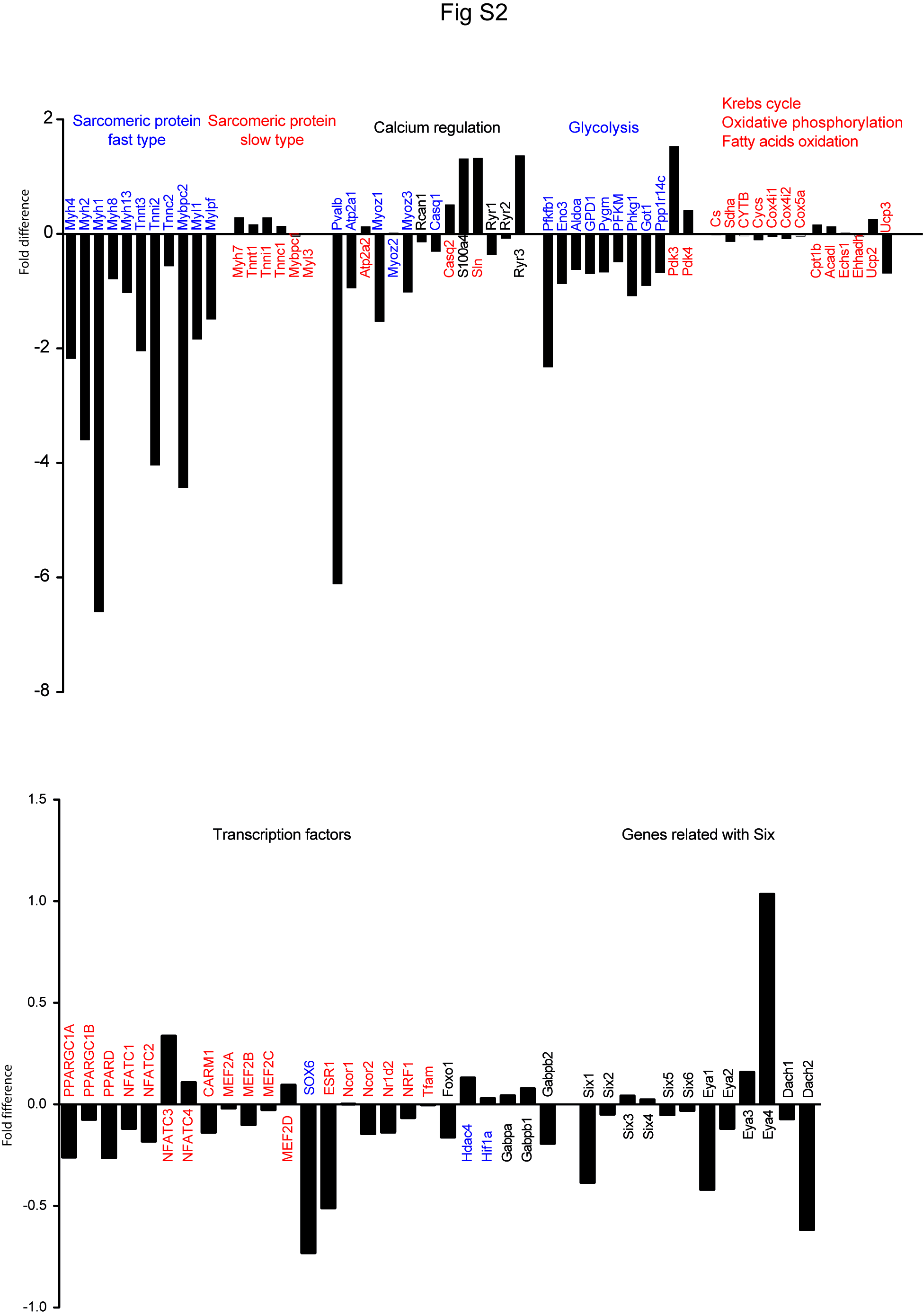

Supplement: Additional file 4: Figure S2. — Affymetrix Microarray analysis showing relative gene expression levels of SOL of 3 months old cSix1 KO mice (n = 3) compared with those of Ctrl mice (n = 3). A set of genes was selected characteristic of slow/fast sarcomeres, glycolysis, mitochondrial oxidation, transcription factors regulating slow/fast phenotype, Six-homeoproteins-related genes. (TIFF 1290 kb) [file 13395_2016_102_MOESM4_ESM.tiff]

Fig S3

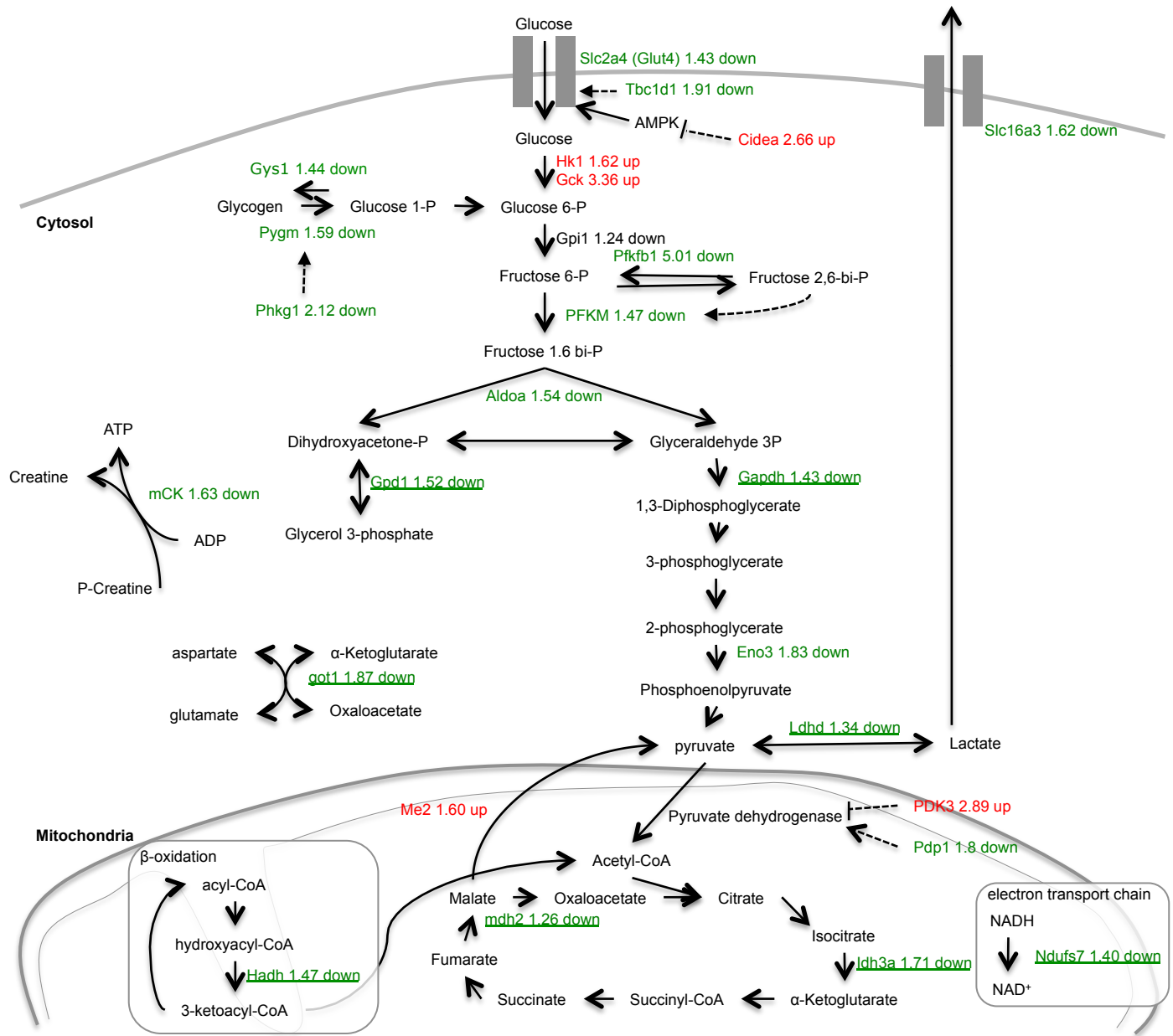

Supplement: Additional file 5: Figure S3. — Gene coding for the glycolytic pathway and the Krebs cycle are represented. Genes whose expression is modified in cSix1 KO are indicated as red (up) or green (down). (PDF 281 kb) [file 13395_2016_102_MOESM5_ESM.pdf]
